# A Review on Passivation Strategies for Germanium-Based Thermophotovoltaic Devices

**DOI:** 10.3390/ma18153427

**Published:** 2025-07-22

**Authors:** Pablo Martín, Ignacio Rey-Stolle

**Affiliations:** Instituto de Energía Solar, ETSI de Telecomunicación, Universidad Politécnica de Madrid, 28040 Madrid, Spain

**Keywords:** germanium, surface passivation, passivation strategies, thermophotovoltaics (TPV), high-efficiency photovoltaics

## Abstract

Interest in germanium electronic devices is experiencing a comeback thanks to their suitability for a wide range of new applications, like CMOS transistors, quantum technology or infrared photonics. Among these applications, Ge-based thermophotovoltaic converters could become the backbone of thermo-electrical batteries. However, these devices are still far from the efficiency threshold needed for industrial deployment, with surface recombination as the main limiting factor for the material. In this work, we discuss the main passivation techniques developed for germanium photovoltaic and thermophotovoltaic devices, summarizing their main advantages and disadvantages. The analysis reveals that surface recombination velocities as low as 2.7 cm/s and 1.3 cm/s have already been reported for *p*-type and *n*-type germanium, respectively, although improving surface recombination velocities below 100 cm/s would result in marginal efficiency gains. Therefore, the main challenge for the material is not reducing this parameter further but developing robust and reliable processes for integrating the current techniques into functional devices.

## 1. Introduction

After a promising start at the early days of electronic research, germanium (Ge) was soon relegated to the background in the development and manufacture of electronic devices, as silicon clearly became the fastest growing and most successful technology in the microelectronic and photovoltaic industries [1,2,3]. Today, germanium still has a dominant role in some applications such as multi-junction solar cells based on III–V compounds that dominate the space market, thanks to their exceptional power-to-weight and power-to-area ratios [4,5]. Nevertheless, the interest in germanium devices is beginning to experience a comeback thanks to the suitability of the material for a wide range of new applications, like high-efficiency CMOS transistors [6,7,8], quantum technology [9], and infrared (IR) photonics [10,11,12].

**Figure 1:**
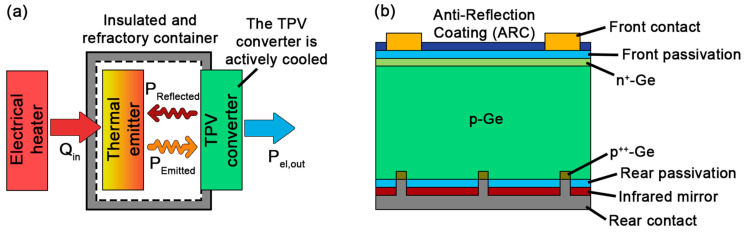
(**a**) Scheme of the basic components of thermophotovoltaic systems and (**b**) example of a typical structure for Ge-based thermophotovoltaic converters.

Focusing on photovoltaic applications, the use of cost-effective Ge-based thermophotovoltaic (TPV) converters seems especially promising for the introduction of this technology in the market [13,14]. This type of device takes advantage of the photovoltaic effect to generate electricity by using semiconductor materials to absorb photons with an energy higher than their bandgap to promote electrons from the valence band to the conduction band [15]. The main peculiarity of TPV systems lies within the irradiance source for the system. While traditional photovoltaic applications use the light radiated by the sun, TPV systems take advantage of the infrared light radiated by incandescent hot energy sources, typically between 1000 °C and 2000 °C (Figure 1a) [16]. Therefore, TPV converters are made using low bandgap semiconductors (such as III–V arsenides or antimonides or germanium) to maximize the fraction of energy absorbed. The second main difference is that, as the energy source (or emitter) and converter are placed in close proximity, TPV devices usually include dielectric or metallic rear infrared mirrors to return the out-of-band energy back to the emitter, reducing the final losses of the system (Figure 1b). This proximity also translates into large photogenerated currents, comparable to those reached in concentration photovoltaics.

Over the last years several new applications have been proposed for TPV systems, pushing the development of the technology [16,17,18,19,20,21,22,23,24,25,26,27,28,29,30]. Among these novel applications, the development of energy storage systems through the use of TPV thermal batteries [28,29,30] stands out as one of the most promising technologies. According to the United Nations goals for sustainable development, by the year 2030, 77% of the energy consumed in the world should be produced using intermittent or variable renewable energies (VREs) [31], such as solar, hydro, or wind energy. However, to transform these VRE sources into fully dispatchable sources, a massive ramping up of electricity storage capacity will be needed [31,32]. In this context, thermal batteries are considered a high-potential technology that could make these objectives possible, thanks to their nominally cheap and energy-dense energy storage materials, with TPV devices an optimal converter for transforming the stored energy back into electricity [13,28,29]. Most of the developments in the context of record-performing TPV converters for thermal batteries have been based on III–V based single-junction or multi-junction devices [33,34,35,36], with record-performing converters already achieving efficiencies well over 40% [33,34]. However, the elevated cost and scarcity associated with these materials could become an insurmountable hindrance for the development of the production volumes needed for field integration [32].

Under these conditions, Ge-based TPV converters are beginning to stand out as a cost-effective alternative to enable the high-volume production needed for the acceptance of the technology in the market, as it is a moderately priced and widely available material, which is already available in the industry of space photovoltaic cells [5,37,38,39]. In fact, germanium was considered a promising candidate for TPV converters since the very beginning of the technology [40], and several authors have recently published new insights on the development of devices using this material [13,14,41,42,43,44]. As a result, Ge-based TPV converters’ performance has been recently pushed over the 20% barrier [13]. However, this value still lies below the necessary threshold for industrial deployment, as, to become market worthy, Ge TPV efficiencies must be further improved to approach at least 30% [32]. Recent analysis on the design of record-performing Ge-based TPV converters pinpoints surface recombination as the main performance-limiting factor in these devices [14], as achieving surface recombination velocities around 100 cm/s seems to fulfill this goal. Nevertheless, due to the high current densities expected in TPV systems (i.e., short-circuit current is usually well over 5 A/cm^2^), good passivation alone is not enough, as high contact quality and low series resistance are critical to reduce any power losses in the device. Moreover, in the case of the rear passivation, any technique employed has to be compatible with a highly reflective infrared mirror. Therefore, understanding the current trends and results reported for germanium surface passivation techniques is critical to achieve these objectives, as a scheme capable of fulfilling all these goals is yet to be implemented.

In this work, we go through the available literature for Ge-based surface passivation strategies, briefly describing the results obtained for each technique developed for the material in terms of their surface recombination velocity (*S_eff_*) and contact quality, identifying promising approaches for their inclusion in improved Ge-based TPV converters.

## 2. Characterization of the Surface Passivation

The quality of the surface passivation for a given material has a major influence on its effective minority carrier lifetime (*τ_eff_*), which can be expressed as follows [45]:

(1) $$\frac{1}{\tau_{eff}}\;=\;\frac{1}{\tau_{Rad}}\;+\;\frac{1}{\tau_{Aug}}\;+\;\frac{1}{\tau_{SRH}}\;+\;\frac{1}{\tau_{Surf}}$$

where *τ_Rad_*, *τ_Aug_*, *τ_SRH_* and *τ_Surf_* are the carrier lifetimes related to radiative, Auger, Shockley–Read–Hall (SRH) and surface recombination mechanisms, respectively.

For high-quality materials, where the effect of bulk recombination mechanisms (i.e., radiative, Auger and SRH) is secondary, higher *τ_eff_* values are always related to better surface passivation. This parameter can be determined using different methods. However, for indirect bandgap semiconductors, the most commonly used characterization technique is through measuring the photoconductance decay (PCD) in the material [46]. This technique is usually used for silicon but can be adapted to germanium [47]. Alternatively, surface passivation quality can also be characterized by its effective surface recombination velocity (*S_eff_*). For low *S_eff_* values, these two parameters can be related as follows [48]:

(2) $$S_{eff}=\left(\frac{1}{\tau_{eff}}\;-\;\frac{1}{\tau_{bulk}}\right)\frac{W}{2}$$

where *τ_bulk_* is the carrier lifetime corresponding to the sum of all the contributions of the bulk recombination mechanisms, as stated before, and *W* is the sample thickness.

For high-quality materials, bulk recombination (*τ_bulk_*) can be considered negligible, and, thus, the corresponding term in Equation (2) can be ignored. Under these assumptions, the PCD method described above can be used to estimate an upper bound for *S_eff_*. Similarly, a slightly more complex but more accurate method can be used to discriminate bulk and surface recombination by measuring several samples with varying thicknesses and identical bulk and surface properties [49]. Alternatively, these parameters can be obtained using the so-called corona-lifetime method [50]. In the vast majority of works summarized in the following section, these approaches are used to extract *S_eff_*.

Moreover, a recent work in the matter analyzes the impact that different recombination mechanisms have on the performance of Ge-based TPV converters [14]. According to the reported results, the impact of bulk recombination mechanisms is secondary in state-of-the-art devices. Instead, surface recombination clearly limits the efficiency of the converters, remarking the importance of improving this parameter if germanium is to be used in commercial TPV applications.

## 3. Germanium Surface Passivation Techniques

**Figure 2:**
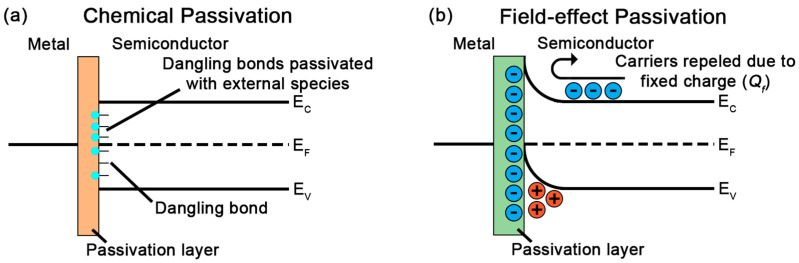
Illustration of the (**a**) chemical passivation and (**b**) field-effect passivation methods.

To counteract the impact that poor surface quality has on the final performance of a photovoltaic device, an adequate passivation of the surfaces and interfaces of the devices should be achieved. The most commonly used passivation techniques usually involve the deposition of one (or several) passivation layers. These layers are usually selected to fulfill two different objectives:

1. Reduce the density of defects (*D_it_*) present at the semiconductor surface through chemical passivation of the interface (Figure 2a). In this way, different species are used to complete the dangling bonds found at the limit of the semiconductor lattice, and the most common are H^−^, S^−^, Cl^−^ and N^−^ [51,52,53].
2. Reduce the number of minority carriers reaching the semiconductor surface, effectively reducing the rate of the recombination processes, through the inclusion of an internal electric field under the passivated surface that repels the charge associated with the corresponding minority carrier [51,53]. This effect can be achieved by either including a heavily doped layer in the surface region (usually referred to as back surface field or BSF) or by including a passivation layer with a fixed charge (*Q_f_*), inducing band bending near the surface (usually referred to as field-effect passivation) (Figure 2b).

In this text, examples of all these techniques are discussed. Nevertheless, it is important to note that, to further reduce the *D_it_* and achieve their full potential, most passivation layers have to be complemented with pre-deposition surface cleaning treatments, interlayers, and/or other post-deposition treatments or annealing processes. Thus, in the following sections, we will provide information not only about the passivation layers used for each technique but also about the pre- and post-treatments necessary to achieve maximum quality. Moreover, as stated above, due to the relatively high current densities expected in TPV devices, any passivation layer included in them should be adequate to form a high-quality electrical contact. Therefore, we will also provide comments on the limitations and opportunities for each treatment to achieve high-quality contacts (and thus, low series resistance). Finally, we will include some notes regarding the suitability of each technique to develop a high-quality rear infrared mirror, vital in high-efficiency TPV devices.

### 3.1. Germanium Oxides

Contrary to silicon, native germanium oxides (GeO_x_) do not typically result in a high-quality interface, as GeO_x_ is a poor oxide that is thermally unstable and soluble in water. Thus, as will be discussed later, native oxides are usually removed using de-ionized (DI) water, wet-chemical processes or plasma exposition prior to the formation of the passivation layer [54], independently of the techniques and materials used.

However, it has been proven that intentionally growing oxides (GeO_2_) through thermal oxidation results in relatively good passivation layers, thanks to a heavy reduction of *D_it_* in the Ge/GeO_2_ interface [55,56,57,58]. Table 1 describes the best result obtained in the context of germanium photovoltaic cells. As can be seen, Chen et al. achieved *S_eff_* up to 70 cm/s on *p*-type Ge by combining a cleaning process based on acetone (to remove the organic contaminants of the surface) and HF (to remove native oxides) with a 47 nm thick GeO_2_ layer formed at 500 °C [58]. As will be discussed later, other techniques achieve lower surface recombination rates, and the high temperatures needed in the oxidation step might result in the unwanted diffusion of contaminant species into the germanium bulk if no special precautions are taken. However, it is important to note the potential of this method, thanks to its simplicity, its suitability to form interlayers in other passivation schemes, and the possibility of using photolithography to easily remove part of the passivating oxide layer to create high-quality contact regions.

**Table 1:** Results of relevant passivation techniques for TPV applications based on GeO_2_ layers.

Substrate	Cleaning	Passivation	*S_eff_* [cm/s]	Reference
p-Ge (1–3 Ω·cm) 60–130 μm	Ex situ: Acetone + DI H_2_O + HF	GeO_2_ by thermal oxidation at 500 °C	70	Chen 2013 [58]

### 3.2. Aluminum Oxides

A more common approach is through the use of high-κ dielectrics, such as aluminum oxides (Al_2_O_3_) [59,60,61,62,63,64]. These layers usually improve passivation through the inclusion of a fixed charge (*Q_f_*) near the surface, leading to the formation of an electric field at the interface rather than a decrease in *D_it_.* Focusing on the studies carried out for photovoltaic devices, summarized in Table 2, Berghuis et al. reported *S_eff_* of 170 cm/s for *p*-type Ge by using atomic layer deposition (ALD) Al_2_O_3_ nanolayers, annealed at an optimal temperature of 425 °C after cleaning the surface with an HF wet-chemical process [60]. This value was further improved to just 2.7 cm/s by the same authors one year later, by including a 1.7 nm thick a-Si:H interlayer deposited by plasma-enhanced chemical vapor deposition (PECVD), followed by a plasma-enhanced ALD (PEALD) Al_2_O_3_ layer, and reducing the annealing temperature to adapt it to the new structure [61]. This heavy reduction in *S_eff_* between these two works can be related to an important increase in *Q_f_* due to the a-Si:H interlayer included in the second scheme (resulting in better field-effect passivation), as well as the formation of a silicon oxide layer between the Al_2_O_3_ and a-Si:H layers (improving the chemical passivation of the interface). Moreover, the deposition technique used for the Al_2_O_3_ layer also plays an important role, as using thermal ALD (such as the one employed in the first work) instead of plasma-enhanced ALD resulted in a substantially lower *Q_f_* due to slightly poorer interface properties [61]. This underlines the great importance of controlling the interface properties for achieving highly passivated surfaces in germanium.

Similarly, the importance of selecting the correct pre- and post-treatments is highlighted in the results reported by Isometsä et al. soon after for *n*-type Ge wafers [62]. In this work, for the same passivation layer deposited under the same conditions, two different values for *S_eff_* of 6.55 cm/s and 11.0 cm/s are obtained for a HCl cleaning combined with an annealing under N_2_ ambient and for an HF cleaning combined with an annealing under forming gas (FG; 95% N_2_ + 5% H_2_) ambient, respectively. This can be related to the different treatments used, leading to a change in the interface properties and *Q_f_*, which results in an increased impact of the electric field formed at the interface.

It is important to note that, as reported by the works just discussed, in germanium the passivation achieved by Al_2_O_3_ layers is usually related to the trapping of a negative *Q_f_* found near the surface of this layer [61,62]. This means that Al_2_O_3_ layers are better suited to passivate *p*-type Ge and lightly doped *n*-type Ge but unable to provide an adequate passivation for highly doped *n*-type Ge. To solve this problem, Theeuwes et al. presented a modified passivation layer by combining a PECVD PO_x_ layer with a PEALD Al_2_O_3_ layer, obtaining a slightly higher *S_eff_* of 8.9 cm/s, in this case related to a positive *Q_f_* [63]. Moreover, during the deposition of the passivation layers, the exposure of the device to an O_2_ plasma heavily contributes to removing any native oxide from the Ge surface. Also, this value is obtained at relatively low annealing temperatures of 250 °C, which further simplifies its fabrication process.

Finally, other works propose the use of alternative, simpler deposition methods for the Al_2_O_3_ passivation layer, such as e-beam evaporation [64]. However, this type of approach results in much higher *S_eff_* values (~1000 cm/s) due to a decrease in the quality of the layer and their associated *Q_f_*. Nevertheless, the previous work also reported that the use of a passivation scheme based on Al_2_O_3_ layers results in a high-quality infrared mirror in the back of the devices, with out-of-band reflectance approaching 98%. This makes the material a specially promising approach for TPV converters, as it is able to provide both an excellent surface passivation and an infrared mirror at the same time. Moreover, as these layers can be selectively removed using photolithography and a chemical etch to form point contacts along the rear surface of the converter [64], they can also achieve the objective of forming a high-quality contact that helps keep the series resistance of the device as low as possible.

**Table 2:** Results of relevant passivation techniques for TPV applications based on Al_2_O_3_ layers.

Substrate	Cleaning	Passivation	*S_eff_* [cm/s]	Reference
p-Ge (0.2 Ω·cm) 400 μm	Ex situ: HF (90″) + DI H_2_O	ALD Al_2_O_3_ Annealing 425 °C (10″) under N_2_	170	Berghuis 2021 [60]
p-Ge (1–3 Ω·cm) 150–304 μm	Ex situ: HF (90″) + DI H_2_O	PECVD a-Si:H/PEALD Al_2_O_3_ Annealing 325 °C (10″) under N_2_	2.7	Berghuis 2021 [61]
n-Ge (18–25 Ω·cm) 185 μm	Ex situ: HF (90″) + DI H_2_O	ALD Al_2_O_3_ Annealing 400 °C (30″) under FG	11	Isometsä 2021 [62]
n-Ge (18–25 Ω·cm) 185 μm	Ex situ: HCl (90″)	ALD Al_2_O_3_ Annealing 400 °C (30″) under N_2_	6.55	Isometsä 2021 [62]
p-Ge (1–3 Ω·cm) 150 μm	Ex situ: HF (90″) + DI H_2_O In situ: O_2_ plasma	PECVD PO_x_/PEALD Al_2_O_3_ Annealing 250 °C (10″) under N_2_	8.9	Theeuwes 2023 [63]
p-Ge (3 Ω·cm) 140 μm	Ex situ: H_3_PO_4_ (15′) + HCl (60″) + DI H_2_O	e-beam Al_2_O_3_ Annealing 400 °C (5″) under N_2_	1000 *	Martín 2023 [64]

* Extracted from EQE and I–V fitting of single-junction Ge TPV converters.

### 3.3. Silicon-Based Passivation

Many authors have focused their efforts on the development of passivation layers based on silicon compounds, as these techniques usually achieve very low surface recombination velocities on germanium and, for the most part, are analogous to the ones used in silicon devices [43,65,66,67,68,69,70,71,72,73,74,75,76,77,78,79,80,81]. The main results obtained in the context of germanium photovoltaics are summarized in Table 3.

For example, Fernández reported *S_eff_* around 55 cm/s for a silicon nitride (SiN_x_) layer grown on a PECVD reactor [68]. This value is mainly related to a significant reduction of *D_it_* in the germanium surface, although a moderate negative *Q_f_* is also observed when including this layer. More recently, Liu et al. [77] reported an almost identical *S_eff_* of 58 cm/s for a similar PECVD SiN_x_ layer. This value was further reduced to 17.5 cm/s for a combination of the previous PECVD SiN_x_ layer and a PEALD Al_2_O_3_ layer comparable to the ones discussed in the previous section. Once again, this improved passivation can be related to the effect of the extra negative *Q_f_* added by the Al_2_O_3_ interlayer. However, despite these results, SiN_x_ might not be the best option for Ge-based thermophotovoltaic devices, as these layers are difficult to etch and, thus, do not facilitate the formation of the high-quality contact needed [68].

For this reason, most authors focus their efforts on other silicon-based compounds such as hydrogenated amorphous silicon carbide (a-Si_x_C_1−x_:H), which also takes advantage of the chemical passivation properties of the layer to obtain lower *D_it_* in the germanium surface, although, once again, it has also been reported to trap a small positive *Q_f_* [72]. Following this approach, the works of Fernández [68], Janz et al. [69], Jimenez et al. [70,71] and Martín et al. [72] are worth mentioning. In the case of Fernández, *S_eff_* between 400 cm/s and 45 cm/s were achieved for the same PECVD a-Si_x_C_1−x_:H layers on wafers with different doping levels [68]. A similar effect, where *S_eff_* depends on the wafer dopant concentration, is observed in the work of Janz et al., where a PECVD a-Si_x_C_1−x_:H passivation layer is combined with a stoichiometric PECVD a-SiC:H layer (used as an interlayer and infrared mirror), obtaining *S_eff_* between 53 cm/s and 17 cm/s [69]. On the one hand, the improvement in passivation observed between the works of Fernández and Janz can be related to an increase in the interface quality thanks to the a-SiC:H interlayer added in the former work. On the other hand, the exact cause of the decrease in the recombination velocities with lower dopant concentrations needs to be studied in detail, but a decrease in the density of the defects or a change in the band structure near the surface region could explain these differences. Alternatively, as dopant concentration decreases, the same *Q_f_* would translate into a higher electric field, which would be more effective in reflecting undesired carriers. These assumptions are further reinforced by the estimations carried out by Fernández and coworkers [68] and Weiss et al. [79], as the *S_eff_* of the layer increased, respectively, to around 1000 cm/s and 900 cm/s when extracting the value from the I–V and EQE curves of a finished photovoltaic cell. This increase can be related to the added thermal load associated with other steps of the fabrication process, resulting in a degradation of the interface and passivation layers that might affect both their *D_it_* and *Q_f_*.

This passivation scheme was later studied in deeper detail in the works of Jimenez et al. [70], who reported the lowest *S_eff_* for a-Si_x_C_1−x_: H-based passivation with a value of just 14.5 cm/s for a PECVD a-Si_x_C_1−x_:H/a-SiC:H stack pre-treated with wet chemical and plasma cleaning methods and annealed using laser pulses. Similar to what happened in the works of Isometsä et al. for Al_2_O_3_ layers, this increase in the passivation quality can be tied to the optimization of the pre- and post-deposition treatments, leading to better interface quality. Following a slightly different approach, Martín et al. reported a similar *S_eff_* of 18 cm/s for a PECVD a-SiC:H/ALD Al_2_O_3_ stack annealed at 400 °C [72]. In this case, the Al_2_O_3_ provides an adequate field-effect passivation thanks to its fixed charge, while, once again, the a-SiC:H layer is used as an interlayer to improve the interface properties and the out-of-band reflectance of the infrared mirror. However, the a-SiC:H layer used in this scheme is intentionally kept as thin as possible, as it is observed that thicker interlayers lead to the trapping of a positive *Q_f_* on their interface, which screens the negative *Q_f_* added by the Al_2_O_3_ passivation layer and results in higher *S_eff_* [72]. It is also important to note that the two former works could also take advantage of a lower dopant concentration to increase the impact of the field-effect passivation and achieve higher *S_eff_* values. In any case, these schemes also present a promising approach for TPV converters, as they exhibit both great passivation and infrared mirror properties. Similarly, it can be argued that the inclusion of a difficult-to-remove a-Si_x_C_1−x_:H layer would result in poor contact qualities, making it inadequate for thermophotovoltaic devices. However, the use of laser-fired contacts (LFCs) could eventually produce series resistance values low enough to enable its use in most applications [82], as demonstrated by the previous works summarized here.

Alternatively, hydrogenated amorphous silicon (a-Si:H) has also proven to be an excellent candidate for germanium passivation, as *S_eff_* as low as 17 cm/s are reported by Posthuma et al. for a PECVD a-Si:H layer [73], thanks to the great chemical passivation and low *D_it_* achieved by this scheme. Similarly, Konagai et al. report *S_eff_* values around 120 cm/s for the same type of passivation layer but measured on a fully manufactured record-performing photovoltaic cell [75], which, as stated before, is expected to present lower interface and passivation layer quality due to the additional stress induced in the rest of the fabrication steps. Although it might be insufficient by itself, these results highlight the potential of this material to reduce the defect density on the surface of germanium, making it an alternative for the deposition of interlayers in other passivation schemes.

Lastly, the use of intentionally grown silicon oxides (SiO_2_) has also been studied in detail due to their potential for achieving great chemical passivation in germanium. In this case, Fernández et al. report surface recombination velocities around 50 cm/s for a PECVD a-Si:H/PECVD SiO_2_ stack [43], as well as for a PECVD a-Si_x_C_1−x_:H/PECVD SiO_2_ stack [76]. Moreover, the lowest recombination velocities achieved for *n*-type Ge have been reported by Liu et al., who presented a *S_eff_* of 10.1 cm/s for a single PEALD SiO_2_ layer and an even lower *S_eff_* of just 1.3 cm/s for a PEALD SiO_2_/PEALD Al_2_O_3_ stack, both annealed at 400 °C [78]. In this last scheme, the excellent chemical passivation of the SiO_2_ interlayer is combined with the negative *Q_f_* of the Al_2_O_3_ layer to achieve the lower surface recombination velocity reported to date. This highlights once again the potential of both materials to be used in high-quality passivation stacks, as they could also meet the stringent passivation, contact and mirror quality requisites needed in high-efficiency Ge TPV devices.

**Table 3:** Results of relevant passivation techniques for TPV applications based on silicon compounds.

Substrate	Cleaning	Passivation	*S_eff_* [cm/s]	Reference
p-Ge (1.1 Ω·cm) 500 μm	Ex situ: H_2_SO_4_ (10″) + H_3_PO_4_ (10′) + DI H_2_O In situ: H_2_ plasma	PECVD SiN_x_ Annealing 400 °C (30″) under N_2_	~55	Fernández 2010 [68]
n-Ge (5–15 Ω·cm) 175 μm	Ex situ: HCl (90″)	PECVD SiN_x_ Annealing 400 °C (30″) under FG	58	Liu 2023 [77]
n-Ge (5–15 Ω·cm) 175 μm	Ex situ: HCl (90″)	PECVD SiN_x_/PEALD Al_2_O_3_ Annealing 400 °C (30″) under FG	17.5	Liu 2023 [77]
p-Ge (1.1 Ω·cm) 150–500 μm	Ex situ: H_2_SO_4_ (10″) + H_3_PO_4_ (10′) + DI H_2_O In situ: H_2_ plasma	PECVD a-Si_x_C_1−x_:H Annealing 400 °C (30″) under N_2_	45	Fernández 2010 [68]
p-Ge (0.2 Ω·cm) 250 μm	Ex situ: H_2_SO_4_ (10″) + H_3_PO_4_ (10′) + DI H_2_O In situ: H_2_ plasma	PECVD a-Si_x_C_1−x_:H Annealing 400 °C (30″) under N_2_	80	Fernández 2010 [68]
p-Ge (0.03 Ω·cm) 250 μm	Ex situ: H_2_SO_4_ (10″) + H_3_PO_4_ (10′) + DI H_2_O In situ: H_2_ plasma	PECVD a-Si_x_C_1−x_:H Annealing 400 °C (30″) under N_2_	400	Fernández 2010 [68]
p-Ge (0.03 Ω·cm) 150 μm	Ex situ: H_2_SO_4_ (10″) + H_3_PO_4_ (10′) + DI H_2_O In situ: H_2_ plasma	PECVD a-Si_x_C_1−x_:H Annealing 400 °C (30″) under N_2_	1000 *	Fernández 2010 [68]
p-Ge (0.2 Ω·cm) 150–500 μm	In situ: H_2_/Ar plasma	PECVD a-Si_x_C_1−x_:H/a-SiC:H Annealing 400 °C (15″)	17	Janz 2017 [69]
p-Ge (0.1 Ω·cm) 150–500 μm	In situ: H_2_/Ar plasma	PECVD a-Si_x_C_1−x_:H/a-SiC:H Annealing 400 °C (30″)	30	Janz 2017 [69]
p-Ge (0.06 Ω·cm) 150–500 μm	In situ: H_2_/Ar plasma	PECVD a-Si_x_C_1−x_:H/a-SiC:H Annealing 400 °C (30″)	53	Janz 2017 [69]
p-Ge (0.2 Ω·cm) 150 μm	In situ: H_2_/Ar plasma	PECVD a-Si_x_C_1−x_:H/a-SiC:H Annealing 400 °C (30″)	900 *	Weiss 2021 [79]
n-Ge (1 Ω·cm) 180 μm	Ex situ: HCl (180″) In situ: H_2_ plasma	PECVD a-Si_x_C_1−x_:H/a-SiC:H Laser-annealed	14.5	Jimenez 2022 [70]
p-Ge (1.2 Ω·cm) 175 μm	Ex situ: HCl (180″) In situ: H_2_ plasma	PECVD a-SiC:H/ALD Al_2_O_3_ Annealing 400 °C (10″) under FG	18	Martín 2022 [72]
p-Ge (11 Ω·cm) 160–500 μm	Ex situ: DI H_2_O (10′) In situ: H_2_ plasma	PECVD a-Si:H	17	Posthuma 2005 [73]
p-Ge (0.28 Ω·cm) 317 μm	Ex situ: HF + HCl	a-Si:H	~120	Konagai 2022 [75]
p-Ge (1.5 Ω·cm) 145 ± 5 μm	Not disclosed	PECVD a-Si:H/SiO_2_ Laser-annealed	~50	Fernández 2007 [43]
p-Ge (0.9 Ω·cm) 140 μm	Not disclosed	PECVD a-Si_x_C_1−x_:H/SiO_2_ Laser-annealed	50	Fernández 2008 [76]
n-Ge (17–39 Ω·cm) 175 μm	Not disclosed	PEALD SiO_2_ Annealing 400 °C (30″) under N_2_	10.1	Liu 2023 [78]
n-Ge (17–39 Ω·cm) 175 μm	Not disclosed	PEALD SiO_2_/Al_2_O_3_ Annealing 400 °C (30″) under N_2_	1.3	Liu 2023 [78]

* Extracted from EQE and I–V fitting of single-junction Ge TPV converters.

### 3.4. Other Techniques

Table 4 summarizes some examples of other germanium passivation techniques found in the literature. Most of the layers discussed up to this point are based on the principles of field-effect passivation and/or chemical passivation. However, as stated in the introduction of this section, a similar effect can be achieved by the inclusion of a BSF layer. In that regard, Fernández reported results for an Al-based BSF layer, achieving *S_eff_* in the order of 1000 cm/s [68]. Virtually the same passivation results were attained by Martín et al. [83] while optimizing the reflection properties of the Al layer to act as an infrared mirror. In this type of process, an aluminum (Al) contact is evaporated on the rear surface of the *p*-type Ge substrate, which acts as the cell base. Then, this layer is annealed at temperatures high enough to favor the diffusion of part of the Al into the germanium (~400 °C). As Al is a group III element, this forms a heavily doped *p*-type region near the surface, which acts as a BSF layer. Although this technique results in far higher surface recombination velocities than most of the previously discussed in this text, it is also one of the simplest, as the passivation can be achieved during the contact formation, making it adequate for its use in devices where the rear surface passivation is not critical (for example, TPV converters manufactured on highly doped *p*-type substrates). Moreover, this technique could also be easily combined with highly reflective rear metal contacts that act as an infrared mirror, fulfilling the requisite of high out-of-band reflectance [83] critical for TPV devices.

Alternatively, going back to the chemical passivation of the dangling bonds found at the interface, most of the processes discussed in the previous sections used different pre-treatments, cleaning methods, and interlayers to achieve this objective, mainly aiming to saturate the dangling bonds on the germanium surface with hydrogen or oxygen atoms. However, Poelman et al. proposed an alternative chemical passivation method based on covering the surface with different iodine (I_2_) solutions [84]. In this work, *S_eff_* below 1000 cm/s are achieved using I_2_ dissolved in polyvinyl acetate in acetone for highly doped *p*-type Ge. Moreover, the effectiveness of the solution improved over time, as *S_eff_* values around 850 cm/s were measured some hours after the treatment of the surface, when the solution achieved its maximum effectiveness, remaining stable after this point.

The role of III–V compounds for the passivation of germanium devices has also been extensively studied as an alternative method to induce band bending near its surface. In fact, III–V compounds are an established passivation scheme typically used at the front surface of Ge-based bottom cells in multi-junction solar cells for space and concentration photovoltaic applications [5,37]. In this case, instead of a fixed charge or heavily doped region, the band bending near the surface is induced by the difference between the bandgap energy of the germanium bulk and III–V passivation layers. Following this approach, Fernández [68] reported *S_eff_* as low as 10 cm/s for a GaInP layer grown by metal–organic vapor phase epitaxy (MOVPE) aimed for its use in multi-junction space cells and single-junction TPV converters. However, despite the suitability of this method to achieve high-quality, highly passivated contacts, it is important to note that the high temperatures typically needed for the growth of the III–V passivation layers could result in cross diffusion between the germanium and the passivating species. In some cases (mostly on the *n*-type region of a germanium device), this could lead to the formation of contaminated regions or parasitic p-n junctions, which would heavily impact their performance. Thus, the study of low-temperature growing processes for III–V layers on germanium substrates should be studied in detail.

Finally, some authors are beginning to focus their efforts on the development of hetero-junction Ge-based devices, thanks to their compatibility with a-Si [85], micro-crystalline silicon (μc-Si) [86,87], and molybdenum oxide (MoO_x_) [88], among others. However, results are still scarce, as this approach is still in the first steps of its development. Thus, further studies are needed to confirm the potential of germanium hetero-structures for photovoltaic applications.

**Table 4:** Results of relevant passivation techniques for TPV applications based on III–V compounds, BSF layers and other approaches.

Substrate	Cleaning	Passivation	*S_eff_* [cm/s]	Reference
p-Ge (0.1 Ω·cm) 150 μm	Ex situ: H_2_SO_4_ (10″) + H_3_PO_4_ (10′) + DI H_2_O In situ: H_2_ plasma	Al BSF Annealing 380 °C	1000 *	Fernández 2010 [68]
p-Ge (3 Ω·cm) 140 μm	Ex situ: H_3_PO_4_ (15′) + HCl (60″) + DI H_2_O	Al BSF Annealing 400 °C (5″) under N_2_	1000 *	Martín 2023 [83]
p-Ge (0.019 Ω·cm) 160 ± 20 μm	Epi-ready (No extra surface cleaning)	Solution of I_2_ dissolved in polyvinyl acetate in acetone	~850	Poelman 2003 [84]
i-Ge 500 μm	In situ: Arsine pre-exposition in a MOVPE	MOVPE GaInP	~10 **	Fernández 2010 [68]

* Extracted from EQE and I–V fitting of single-junction Ge TPV converters. ** Measured on intrinsic Ge wafers.

### 3.5. Effect of Surface Passivation on TPV Efficiency

**Figure 3:**
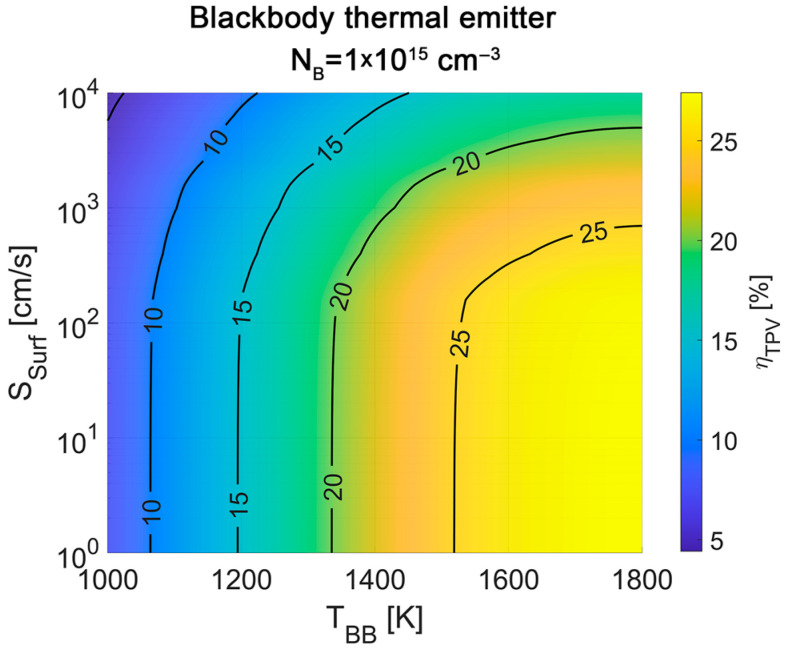
Thermophotovoltaic efficiency estimated as a function of the emitter temperature and the effective surface recombination velocity for a record-performing TPV converter.

To illustrate the effect that a change in *S_eff_* might have on the final performance of a TPV device, an estimation of the TPV efficiency (*η_TPV_*) as a function of the temperature of a blackbody thermal emitter (*T_BB_*) and the effective surface recombination velocity (*S_Surf_*) of the converter was carried out using SILVACO Atlas. The simulation was carried out using as a base the experimental data reported in previous works for record-performing Ge-based TPV converters [13], considering an absorber dopant concentration of *N_B_* = 1 × 10^15^ cm^−3^. The I–V curves and external quantum efficiency (EQE) are fitted to extract the front surface and rear surface recombination rates of the TPV converter. These rates are then gradually minimized to analyze the change in I–V curves and TPV efficiency. The exact details of the simulation model [14] and the method for the calculation of *η_TPV_* [13] can be found in previous works. The results obtained are represented in Figure 3. For the sake of simplicity, the front and rear surface recombination velocities are considered to be equal in the analysis (i.e., the simulations are carried out always considering an identical *S_eff_* for the front and rear surfaces of the device).

According to the simulations, reducing *S_Surf_* from 10,000 cm/s to 100 cm/s translates into an increase up to ~50% in the final efficiency of the converter, with its impact more relevant as *T_BB_* rises. However, further reducing *S_Surf_* below the 100 cm/s threshold seems to translate into only marginal improvements in the performance. Thus, even though recombination velocities reported for germanium are still higher than the ones reported for silicon [89,90], their impact on the final performance of current devices might already be minimal, at least for thermophotovoltaic converters.

## 4. Conclusions

Defects present in the surfaces and interfaces of semiconductors can heavily impact the final performance of Ge-based photovoltaic devices. Thus, if high-efficiency germanium thermophotovoltaic (and photovoltaic) converters are to be developed, specialized high-quality passivation processes are necessary.

In the case of germanium, the reported techniques are relatively similar to those used in silicon, as most focus on removing the native oxide layers and then depositing several types of passivation layers. Among these, the ones based on taking advantage of a combination of chemical passivation and field-effect passivation of the surface seem especially promising, as Al_2_O_3_ and Si-based layers (or stacks) present the lowest effective surface recombination velocities reported for the material, with values of 2.7 cm/s and 1.3 cm/s for *p*-type and *n*-type germanium, respectively.

However, despite the efforts of several groups to improve the current techniques, germanium surface recombination velocity is still substantially higher than the one achieved in silicon devices. Nevertheless, an analysis of the impact that an improvement in this parameter might have on the performance of Ge-based TPV devices reveals that improvements beyond this point might result in only marginal efficiency gains. Thus, the benefits of further decreasing surface recombination in Ge-based photovoltaic devices might be unclear, and efforts should be focused first on integrating reliably and reproducibly the current techniques into functional thermophotovoltaic (and photovoltaic) converters.

In any case, even though achieving surface recombination velocities approaching zero (as the ones currently reported for silicon) seems unlikely and secondary for germanium in the near future, continued research on the topic is still needed. As the processing and manufacturing of Ge-based thermophotovoltaic devices advances, performance could once again become limited by the passivation quality. Therefore, special attention should be paid to developing more advanced passivation techniques for the material that achieve the triple objective of achieving high surface passivation, very low series resistance and highly reflective infrared mirrors. In that regard, it is likely that the use of stacks of passivation layers (especially the ones including Al_2_O_3_ passivation layers and oxide interlayers) will become the preferred choice to achieve these goals, since this type of structure can be easily adapted to the photolithography, annealing or laser processes necessary to form high-quality contacts and dielectric infrared mirrors, critical for thermophotovoltaic (and even photovoltaic) applications, allowing for further improvements in the performance and reliability of future devices.

## Data Availability

No new data were created or analyzed in this study. Data sharing is not applicable to this article.
